# Comparison of cardiovascular magnetic resonance characteristics and clinical prognosis in left ventricular noncompaction patients with and without arrhythmia

**DOI:** 10.1186/s12872-022-02470-7

**Published:** 2022-02-02

**Authors:** Zi-qi Zhou, Wen-chong He, Xiao Li, Wei Bai, Wei Huang, Rui-lai Hou, Yi-ning Wang, Ying-kun Guo

**Affiliations:** 1 Department of Radiology, Key Laboratory of Birth Defects and Related Diseases of Women and Children of Ministry of Education, West China Second University Hospital, Sichuan University, 20# Section 3 South Renmin Road, Chengdu, 610041 China; 2 Research Management Office, West China Second University Hospital, Sichuan University, Chengdu, China; 3 Department of Radiology, Peking Union Medical College Hospital, Chinese Academy of Medical Sciences, Dongcheng District, Peking Union Medical College, No. 1 Shuaifuyuan, Beijing, 100730 China

**Keywords:** Left ventricular noncompaction, Cardiac magnetic resonance, Arrhythmia, Prognosis

## Abstract

**Background:**

Left ventricular noncompaction (LVNC) is a rare type of cardiomyopathy, and one of its clinical manifestations is arrhythmia. Cardiovascular magnetic resonance (CMR) is valuable for the diagnosis and prognosis of LVNC. However, studies are lacking on the use of CMR for LVNC patients with arrhythmia. This study aimed to characterize and compare CMR features and prognosis in LVNC patients with and without arrhythmia.

**Methods:**

Eighty-four LVNC patients diagnosed by CMR were enrolled retrospectively in this study. Clinical data, arrhythmia characteristics, and CMR parameters were collected. Patients were divided into different groups according to the arrhythmia characteristics and CMR manifestations for statistical analysis and comparison. Ventricular tachycardia (VT), ventricular fibrillation (Vf), ventricular flutter (VFL), III° atrioventricular block (III° AVB), Wolff–Parkinson–White syndrome (WPW) and ventricular escape (VE) were defined as malignant arrhythmias and benign arrhythmias included premature ventricular contraction, atrial premature beats, atrial fibrillation, supraventricular tachycardia, supraventricular premature beat, bundle branch block, atrial flutter and sinus tachycardia. The outcome events were defined as a composition event of cardiac death, rehospitalization for heart failure, heart transplantation, and implantation of an implantable cardioverter defibrillator (ICD).

**Results:**

Sixty-seven LVNC patients (79.76%) mainly presented with arrhythmia, including premature ventricular beat (33 patients [27.73%]), bundle branch block (14 patients [11.77%]), electrocardiogram waveform changes (18 patients [15.13%]), and ventricular tachycardia (11 patients [9.24%]). The cardiac function and structure parameters had no significant difference among the nonarrhythmia group, benign arrhythmia group, and malignant arrhythmia group. However, the presence of late gadolinium enhancement (LGE) was higher in the malignant arrhythmia group than in the other two groups (*p* = 0.023). At a mean follow-up of 46 months, cardiac events occurred in twenty-three patients (46.94%). Kaplan–Meier analysis showed that there was no statistically significant difference in prognosis among the nonarrhythmia, benign, and malignant arrhythmia groups, but the patients with arrhythmia and association with LGE + or left ventricular ejection fraction (LVEF) < 30% had a higher risk than patients with LGE- or LVEF > 30% (LGE +, HR = 4.035, 95% CI 1.475–11.035; LVEF < 30%, HR = 8.131, 95% CI 1.805–36.636; *P* < 0.05).

**Conclusions:**

In LVNC patients, the types of arrhythmias are numerous and unrepresentative, and arrhythmia is not the prognostic factor. Arrhythmia combined with presence of LGE or LVEF < 30% is associated with poor prognosis in LVNC patients.

## Background

Left ventricular noncompaction (LVNC) is a rare type of cardiomyopathy that is characterized by the presence of numerous prominent trabeculations and deep intertrabecular recesses connected to the left ventricular cavity. It can exist in isolation or in combination with other congenital heart diseases [1, 2]. The disease may be asymptomatic or have clinical manifestations such as severe heart failure (HF), arrhythmia, systemic thromboembolic events, and sudden cardiac death [3, 4]. Among these clinical manifestations, arrhythmia is one of the most common symptoms in LVNC patients, including ventricular tachycardia (VT), premature ventricular contraction (PVC), and others [5]. Furthermore, the prognosis of LVNC patients is mainly related to the severity of the cardiac structure and function changes. The research by Oechslin and Jenni [6] showed that left ventricular dilation increased the risk of cardiac death. In addition, patients with reduced left ventricular ejection fraction (LVEF) and left ventricular fibrosis are considered to have a higher incidence of adverse cardiovascular events in LVNC patients [7]. Other studies considered that the prognosis of LVNC patients would also be affected by malignant arrhythmias, which can cause severe hemodynamic disturbance in a short time, thus leading to syncope and even sudden death [8–11]. However, the assessments of LVNC in most studies were evaluated by ultrasound, and comparisons between LVNC patients with and without arrhythmia have rarely been described.

Recently, cardiac magnetic resonance (CMR) has become one of the noninvasive examination methods used for the diagnosis of heart disease, including LVNC. CMR with late gadolinium enhancement (LGE) is a reliable technique for detecting myocardial fibrosis in vivo, which is related to the prognosis in patients with LVNC and the occurrence of ventricular arrhythmia [12, 13]. Wu et al. [14] compared the CMR manifestations of hypertrophic cardiomyopathy patients with and without arrhythmias and found differences in the results between the two groups. Additionally, for patients with chronic myocardial infarction, the CMR characteristics and prognosis of malignant arrhythmia are different from those of patients without malignant arrhythmia [15]. CMR is showing increasingly more diagnostic potential in cardiomyopathy, but studies on the assessment of LVNC patients with or without arrhythmia are restricted to a few case series. Therefore, this study aims to compare the differences in the CMR manifestations of LVNC patients with different degrees of arrhythmia, summarize the characteristics of arrhythmia in patients, and further explore the risk factors for prognosis in LVNC patients with arrhythmia.

## Materials and methods

### Study population and design

Patients who underwent standard gadolinium-enhanced CMR scans for cardiac assessment in three health centers between January 2010 and December 2019 were included in the retrospective cohort. The inclusion criteria include patients who were diagnosed with LVNC by CMR [16]: (1) The left ventricular myocardium is composed of two layers, namely, the normal compacted but thin outer myocardium and the significantly thickened noncompact inner myocardium; (2) There are prominent myocardial trabeculations in the noncompacted myocardium and deep intertrabecular recesses communicating with the left ventricle (LV); (3) On the four-chamber slices of the LV, the end-diastolic noncompact/compact (NC/C) ratio > 2.3. The exclusion criteria were as follows: (1) presence of other known coexisting cardiac abnormalities, including congenital heart disease, coronary heart disease, valvular heart disease, hypertrophic cardiomyopathy, dilated cardiomyopathy or other types of cardiomyopathy, and myocarditis; (2) combination with diabetes, liver and kidney insufficiency, tumor, infection, hyperthyroidism, and other diseases that may cause arrhythmia; (3) absence of electrocardiography (ECG) results; (4) poor CMR image quality; and (5) incomplete clinical records. Patients with VT, ventricular fibrillation (Vf), ventricular flutter (VFL), III° atrioventricular block (III° AVB), Wolff–Parkinson–White syndrome (WPW) and ventricular escape (VE) were thought to have malignant arrhythmia, and others were considered benign arrhythmias. Patients were divided into three groups, including the nonarrhythmia group, benign arrhythmia group, and malignant arrhythmia group according to arrhythmia status. Participants were divided into two groups on the basis of decreased ejection fraction, namely, the LVEF < 30% group and LVEF > 30% group. A cross-sectional study was used to compare CMR performance and other data of different groups, and the prognosis of some patients was compared by the retrospective cohort study. This study was approved by the institutional ethics review board of three medical centers, and informed consent was obtained from every patient with LVNC.

### CMR protocol

All gadolinium-enhanced CMR exams were performed using a 3.0 T scanner (Skyra; Siemens Medical Solutions, Erlangen, Germany) with a cardiac phased-array coil. Retrospective ECG-gated cine images were performed using the steady-state free-precession (SSFP) sequence to obtain cine images on the short axis, two-chamber long axis, three-chamber long axis, and four-chamber long axis. The parameters were as follows: 241 mm × 300 mm FOV, 6–8 mm slice thickness, 0 mm slice gap, 44.46 ms repetition time, 1.5 ms echo time, and 60° flip angle. Gadobenate dimeglumine (MultiHance 0.5 mmol/mL; Bracco, Milan, Italy) was injected intravenously with a flow rate of 2.5–3.0 mL/s and a dose of 0.1–0.2 mL per kg of body weight. At the same time, a 20–25 mL saline flush was injected at a 3.0 mL/s flow rate. 10–15 min after the contrast injection, LGE images were obtained using the inversion recovery MOCO sequence. The parameters were as follows: 340 mm × 360 mm FOV, 8 mm slice thickness, 684.00 ms repetition time, 1.04 ms echo time, and 55° flip angle.

### CMR image analysis

**Fig. 1:**
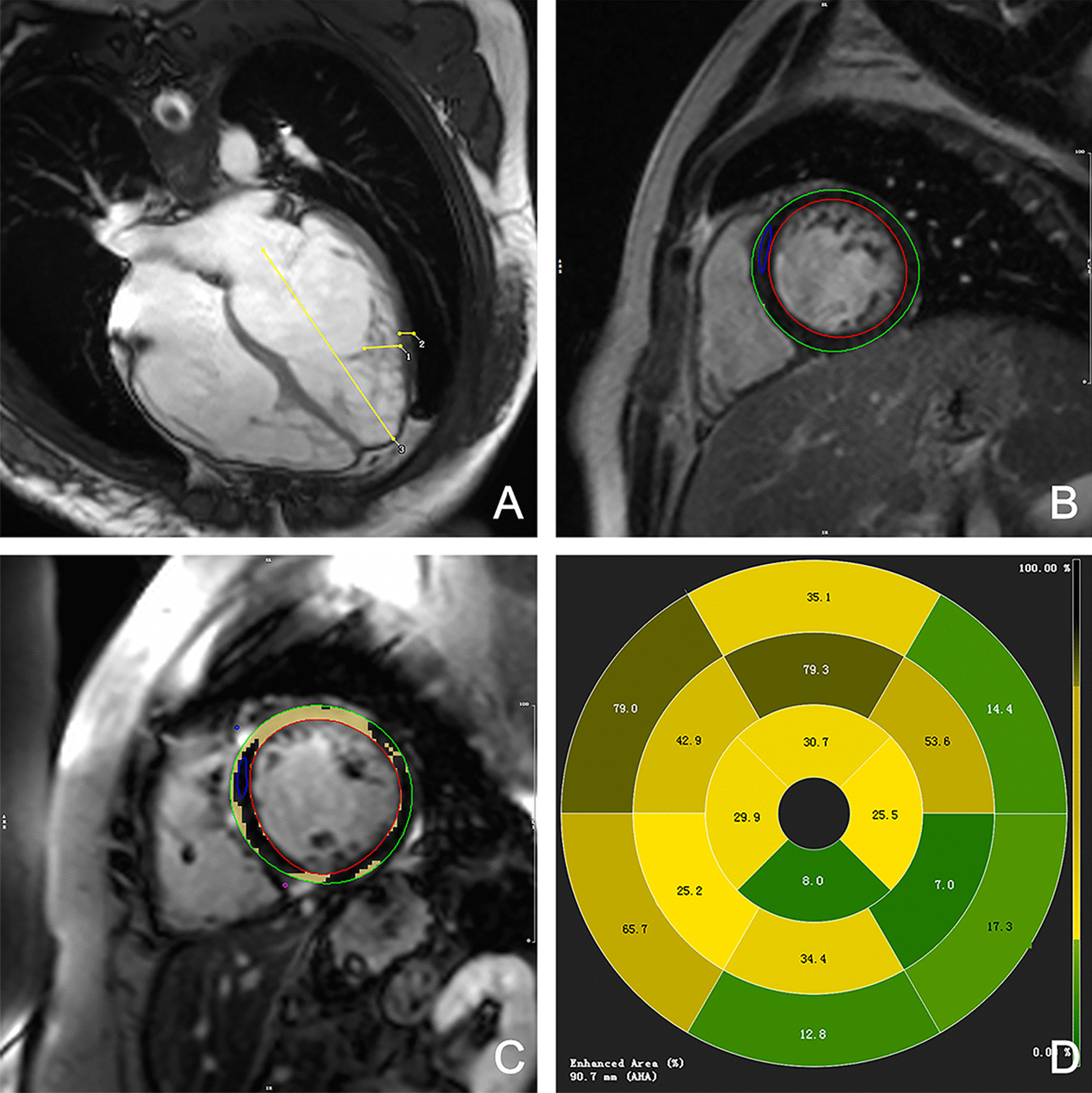
Schematic diagram of partial parameter measurement of CMR. **a** End-diastolic phase of the 4-chamber cine image, line 1 measured the thickness of the noncompacted myocardium, line 2 measured the thickness of the compacted myocardium, line 3 (from mitral orifice to apex) measured the length of the long axis of the left ventricle; **b** LGE-, the red circle represented the endocardial boundarie, green circle represented the epicardial boundarie and the blue circle represented the normal myocardium; **c** LGE +, the yellow blocks represented areas of LGE identified by the software; **d** AHA 17 segment model distributions of LGE

All CMR images were analyzed using the commercially available postprocessing software Cvi42 (Circle Cardiovascular Imaging, Calgary, Canada). Cardiac function parameters, including LVEF, end-diastolic volume (EDV), end-systolic volume (ESV), stroke volume (SV), and myocardial mass were derived from short-axis cine images. The end-diastolic phase of the four-chamber cine images was used to measure the length of the long axis of the LV. In addition, the thicknesses of the noncompacted and compacted myocardium were measured at the places where the noncompaction was significant (Fig. 1a). The number of segments with noncompacted myocardium was calculated using the AHA 17 segment model. The presence and amount of LGE were assessed and quantified on short-axis images, and LGE was deemed present if myocardial enhancement was confirmed on short-axis areas by using a signal intensity threshold of 5SD above the mean signal of the remote normal myocardium (Fig. 1b–d). The end-diastolic and end-systolic phases were defined as those with maximum and minimum visual areas, respectively. The endocardial and epicardial boundaries of all images were manually delineated by a radiologist with at least two years of experience who was blinded to the clinical information. When delineated, the papillary muscles were excluded from the compacted myocardium [17].

### Follow-up

A clinician with no knowledge of the clinical and CMR information of the patients contacted some of the patients or their families by telephone for a standard questionnaire interview after the initial CMR examination. The endpoints of the study were major adverse cardiovascular events (MACEs), including cardiac death, rehospitalization due to HF, heart transplantation, and implantation of an implantable cardioverter defibrillator (ICD). The follow-up duration was determined from the date of the first CMR evaluation to the occurrence of the endpoint. If no endpoint occurred, the follow-up period ended on the date of the telephone questionnaire interview.

### Statistical analysis

Statistical analysis was performed using commercially available software SPSS 23.0 (Chicago, IL, USA). Normality was tested using the Kolmogorov–Smirnov test. Continuous variables were expressed as mean ± standard deviation, and intercohort evaluations were assessed using one-way ANOVA. Categorical variables were presented as counts and frequencies and were assessed using the χ^2^ test or Fisher’s exact test. The survival curve was generated by the Kaplan–Meier method and compared by the log-rank tests. A two-tailed *p* value < 0.05 was considered statistically significant.

## Results

### Baseline characteristics

A total of 84 patients participated in this study. 17 patients (20.24%) had no arrhythmia, 48 patients (57.14%) had benign arrhythmia, and 19 patients (22.62%) had malignant arrhythmia. 51 participants (60.71%) were male, and 33 participants (39.29%) were female. The mean age of patients was 42.87 ± 20.70 years old. 14 patients (16.67%) had a family history of cardiovascular disease. 24 patients (28.57%) had a history of smoking, and 25 patients (29.76%) had a history of drinking. 12 patients (14.29%) had hypertension, and 50 patients (59.52%) presented with the signs and symptoms of congestive HF. Only 5 patients (5.95%) had a thrombus in the LV cavity. Table 1 shows the clinical and demographic characteristics of the study population. There was no significant difference in clinical and demographic characteristics among the three groups (*p* > 0.05).

**Table 1:** Baseline characteristics

Variable	All participants (n = 84)	No arrhythmia (n = 17)	Benign arrhythmia (n = 48)	Malignant arrhythmia (n = 19)	*P* value
Male, n (%)	51 (60.71)	10 (58.82)	26 (54.17)	15 (78.95)	0.171
Age (years)	42.87 ± 20.70	47.71 ± 18.76	44.23 ± 19.93	35.11 ± 23.13	0.149
BMI^a^	24.10 ± 5.48	24.51 ± 5.28	22.99 ± 2.64	26.79 ± 9.61	0.199
Family history of cardiovascular disease, n (%)	14 (16.67)	4 (23.53)	6 (12.50)	4 (21.05)	0.480
History of smoking, n (%)	24 (28.57)	3 (17.65)	17 (35.42)	4 (21.05)	0.269
History of drinking, n (%)	25 (29.76)	4 (23.53)	15 (31.25)	6 (31.58)	0.820
Hypertension, n (%)	12 (14.29)	5 (29.41)	5 (10.42)	2 (10.53)	0.197
Syncope, n (%)	16 (19.05)	3 (17.65)	8 (16.67)	5 (26.32)	0.694
Heart failure, n (%)	50 (59.52)	8 (47.06)	31 (64.58)	11 (57.89)	0.443
NYHA functional class					0.086
0, n (%)	16 (19.05)	5 (29.41)	8 (16.67)	3 (15.79)	
I, n (%)	7 (8.33)	0 (0)	4 (8.33)	3 (15.79)	
II, n (%)	22 (26.19)	2 (11.76)	18 (37.50)	2 (10.53)	
III, n (%)	35 (41.67)	8 (47.06)	17 (35.42)	10 (52.63)	
IV, n (%)	4 (4.76)	2 (11.76)	1 (2.08)	1 (5.26)	
Thrombo-embolic events, n (%)	5 (5.95)	1 (5.88)	2 (4.17)	2 (10.53)	0.592
Medications					
Aldosterone antagonists, n (%)	55 (65.48)	12 (70.59)	32 (66.67)	11 (57.89)	0.701
β-Blockers, n (%)	53 (63.10)	7 (41.18)	33 (68,75)	13 (68.42)	0.111
ACEI/ARB, n (%)	32 (38.10)	4 (23.53)	17 (35.42)	11 (57.89)	0.089
Loop diuretics, n (%)	52 (61.90)	13 (76.47)	31 (64.58)	8 (42.11)	0.089
Amiodarone, n (%)	6 (7.14)	1 (5.88)	3 (6.25)	2 (10.53)	0.845

Values are mean ± SD or n (%)

^a^Calculated as weight in kilograms divided by height in meters squared

### Arrhythmia characteristics

Among the 84 patients with LVNC, 67 patients (79.76%) had arrhythmias of varying degrees, 35 patients (41.67%) presented primarily with one type of arrhythmia, and 32 patients (38.09%) had multiple types of ECG abnormalities. Among LVNC patients with arrhythmia, ventricular arrhythmia was the most common. Among all arrhythmias that occurred, PVC occurred in 33 patients (27.73%), VT occurred in 11 patients (9.24%), and the ECG of 18 patients (15.13%) showed ST-T segment changes or abnormal Q waves. Table 2 shows the other arrhythmia characteristics.

**Table 2:** Arrhythmia characteristics of 84 LVNC patients

Arrhythmia manifestations	LVNC patients (n = 84)
Normal result of ECG, n (%)	17 (20.24)
Single type of arrhythmia, n (%)	35 (41.67)
Combined with multiple arrhythmias, n (%)	32 (38.09)

Types of arrhythmias	Number of cases (n = 119)
PVC, n (%)	33 (27.73)
ECG waveform changes, n (%)	18 (15.13)
CLBBB/CRBBB, n (%)	14 (11.77)
APB, n (%)	11 (9.24)
VT, n (%)	11 (9.24)
Af, n (%)	9 (7.57)
Sinus tachycardia, n (%)	7 (5.88)
AVB, n (%)	5 (4.20)
SVT, n (%)	4 (3.36)
SVES, n (%)	2 (1.68)
VE, n (%)	3 (2.52)
AFL, n (%)	1 (0.84)
WPW, n (%)	1 (0.84)

Values are n (%)

### CMR findings

The detailed CMR characteristics of patients with LVNC are reported in Table 3. The mean LVEF, EDV, and ESV were (23.99 ± 14.20) %, (248.09 ± 105.21) mL, and (189.34 ± 100.62) mL, respectively. The mean number of noncompacted segments per patient was 5.94 ± 1.91, and the mean NC/C ratio was 3.25 ± 1.18. The mean SV, long-axis diameter, sphericity index, and myocardial mass of the LV were (58.75 ± 24.31) mL, (70.40 ± 16.54) mm, 1.46 ± 0.62, and (113.94 ± 45.59) g, respectively. In addition, the number of patients with significantly decreased LVEF (LVEF < 30%) in each group was 9, 38, and 11, respectively. However, there was no significant difference in these CMR parameters among the three groups (*p* > 0.05).

**Table 3:** Cardiovascular magnetic resonance characteristics in LVNC patients with and without arrhythmia

Variable	All participants (n = 84)	No arrhythmia (n = 17)	Benign arrhythmia (n = 48)	Malignant arrhythmia (n = 19)	*P* value
LVEF (%)	23.99 ± 14.20	30.32 ± 13.65	22.95 ± 13.71	20.96 ± 14.92	0.105
LVEDV (mL)	248.09 ± 105.21	253.41 ± 83.71	248.29 ± 108.44	242.81 ± 118.54	0.956
LVESV (mL)	189.34 ± 100.62	182.02 ± 84.18	192.04 ± 103.50	189.05 ± 111.08	0.941
SV (mL)	58.75 ± 24.31	71.39 ± 28.47	56.25 ± 21.14	53.76 ± 25.38	0.05
LV long-axis diameter (mm)	70.40 ± 16.54	71.70 ± 11.31	71.81 ± 16.71	65.65 ± 19.69	0.368
LV Sphericity index^#^	1.46 ± 0.62	1.39 ± 0.51	1.39 ± 0.64	1.70 ± 0.65	0.162
Myocardial mass (g)	113.94 ± 45.59	120.98 ± 26.38	117.41 ± 52.91	98.87 ± 36.57	0.114
NC/C ratio	3.25 ± 1.18	3.02 ± 0.86	3.34 ± 1.29	3.21 ± 1.15	0.635
Number of non-compacted segments	5.94 ± 1.91	5.29 ± 1.76	6.06 ± 2.04	6.21 ± 1.69	0.288
LGE, n (%)	24 (28.57)	1 (5.88)^a^	14 (29.17) ^a, b^	9 (47.37) ^b^	0.023
LVEF < 30%, n (%)	58 (69.05)	9 (52.94)	38 (79.17)	11 (57.89)	0.065

Values are mean ± SD or n (%)

^#^Calculated as: end-diastolic volume/([long-axis diameter^3^ × Π]/6)

^a,b^The same letter represented no statistically significant difference between groups

In addition, a total of 24 patients (28.57%) with LVNC showed LV LGE. Among the patients without arrhythmia, only one patient (5.88%) had LGE. LGE occurred in 14 patients with benign arrhythmia (29.17%) and in 9 patients with malignant arrhythmia (47.37%). There was a significant difference in terms of the number of patients with LGE among the three groups (*p* = 0.023). Patients with malignant arrhythmia had a significantly higher incidence of LGE than patients without arrhythmia (47.37% vs. 5.88%, *p* < 0.05). In addition, there were 0 (17, 0%), 12 (48, 25%), and 7 (19, 36.84%) patients with LGE positive and LVEF < 30% in the three groups, respectively.

### Follow-up

**Fig. 2:**
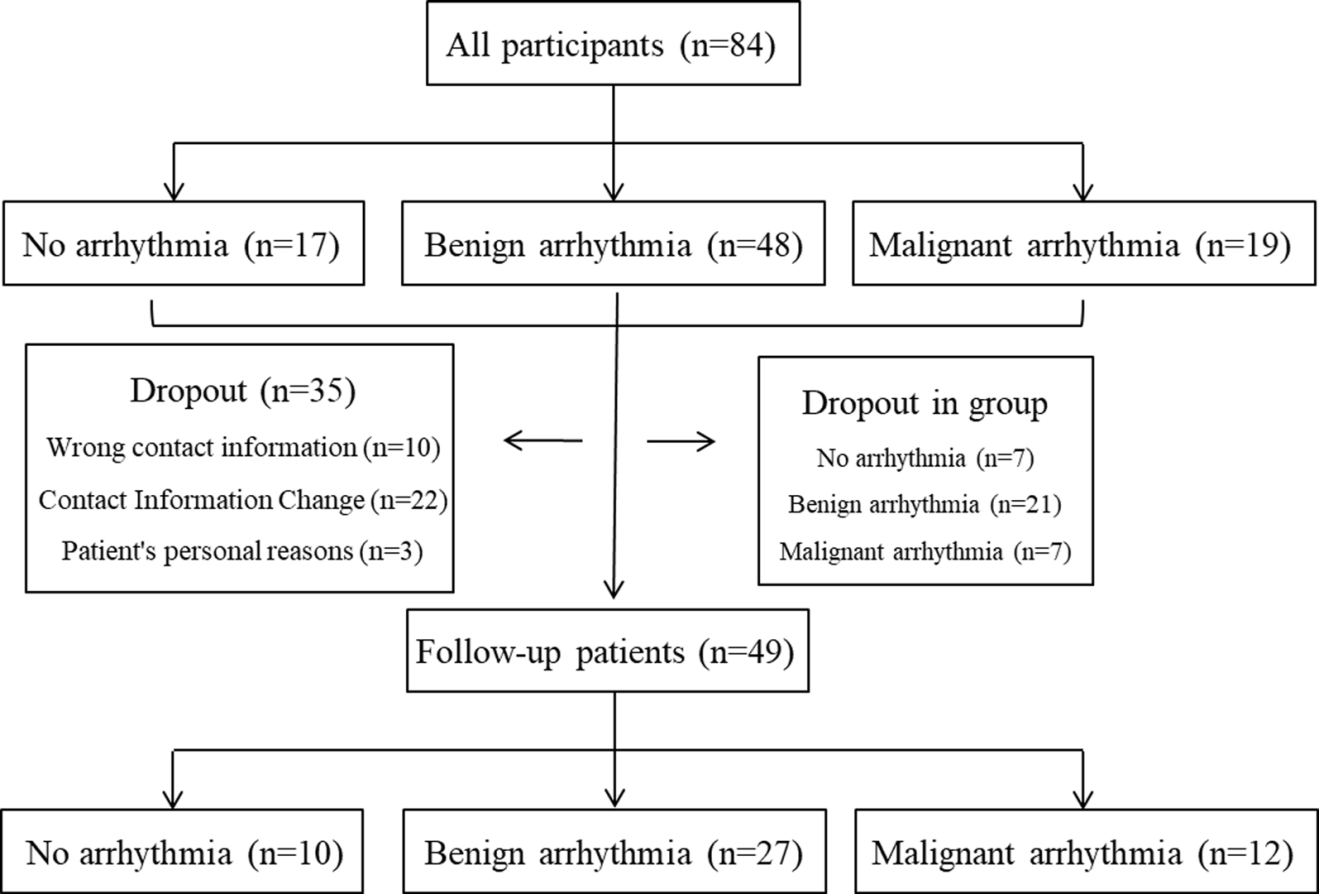
Patient follow-up flow diagram. Due to incorrect contact information, change of contact information, and patients' personal wishes, and others, 49 patients were finally followed up. There were 10 patients in the non-arrhythmia group, 27 in the benign arrhythmia group and 12 in the malignant arrhythmia group

Among the 49 patients who were followed up, 10 patients had no arrhythmia, 27 patients had benign arrhythmia, and 12 patients had malignant arrhythmia (Fig. 2). The period of follow up is 19–73 months and the average length of follow-up from diagnosis was (46.02 ± 26.60) months. During the follow-up period, 11 patients (22.45%) died of cardiac causes, 1 patient (2.04%) underwent heart transplantation, and 3 patients (6.12%) had ICD implanted. Another 8 patients (16.33%) were readmitted for heart problems. Hence, the total cardiac event rate was 46.94%. Table 4 shows the contribution of each group of patients to adverse cardiovascular events.

**Table 4:** Incidence of adverse cardiovascular events

Adverse cardiovascular events	LVNC patients (n = 49)	No arrhythmia (n = 10)	Benign arrhythmia (n = 27)	Malignant arrhythmia (n = 12)
Cardiac death	11 (22.45)	1 (10.00)	6 (22.22)	4 (33.33)
Rehospitalization because of heart failure	8 (16.33)	2 (20.00)	4 (14.82)	2 (16.67)
Heart transplantation	1 (2.04)	1 (10.00)	0 (0.00)	0 (0.00)
Installation of pacemaker	3 (6.12)	110.00)	1 (3.70)	1 (8.33)
Total	23 (46.94)	5 (50.00)	11 (40.74)	7 (58.33)

Values are n (%)

**Fig. 3:**
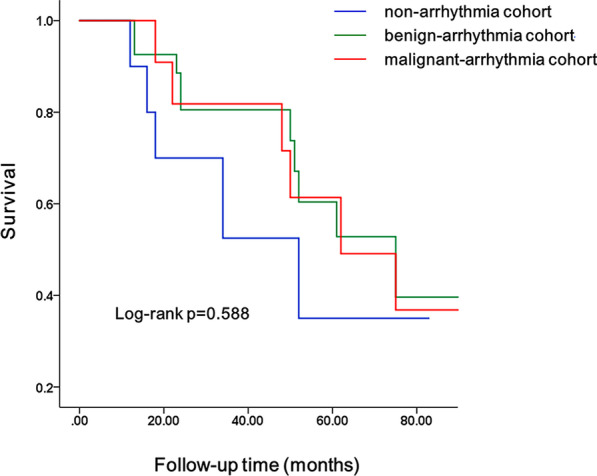
K–M survival curve among three groups: nonarrhythmia, benign, and malignant arrhythmia. There was no significant difference in the incidence of adverse events among the three groups in LVNC patients without arrhythmia, benign arrhythmia and malignant arrhythmia

In the nonarrhythmia cohort, adverse events occurred in five patients. The number of adverse events in the benign and malignant arrhythmias cohorts was 11 and 7, respectively. However, Kaplan–Meier analysis showed that there was no significant difference in outcome for adverse cardiovascular events among the three cohorts (Fig. 3).

**Fig. 4:**
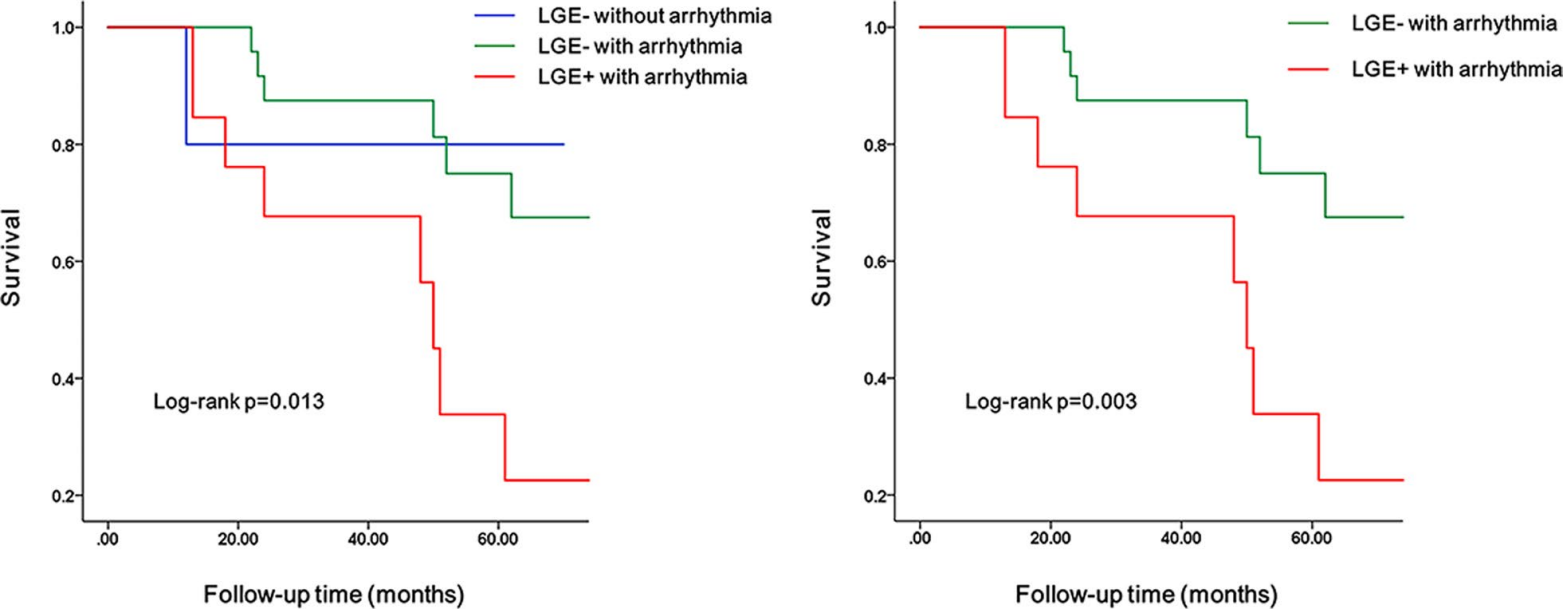
K–M survival curve incorporating LGE into grouping conditions. Kaplan–Meier analysis among three cohorts: LGE- without arrhythmia vs. LGE- with arrhythmia vs. LGE + with arrhythmia

**Fig. 5:**
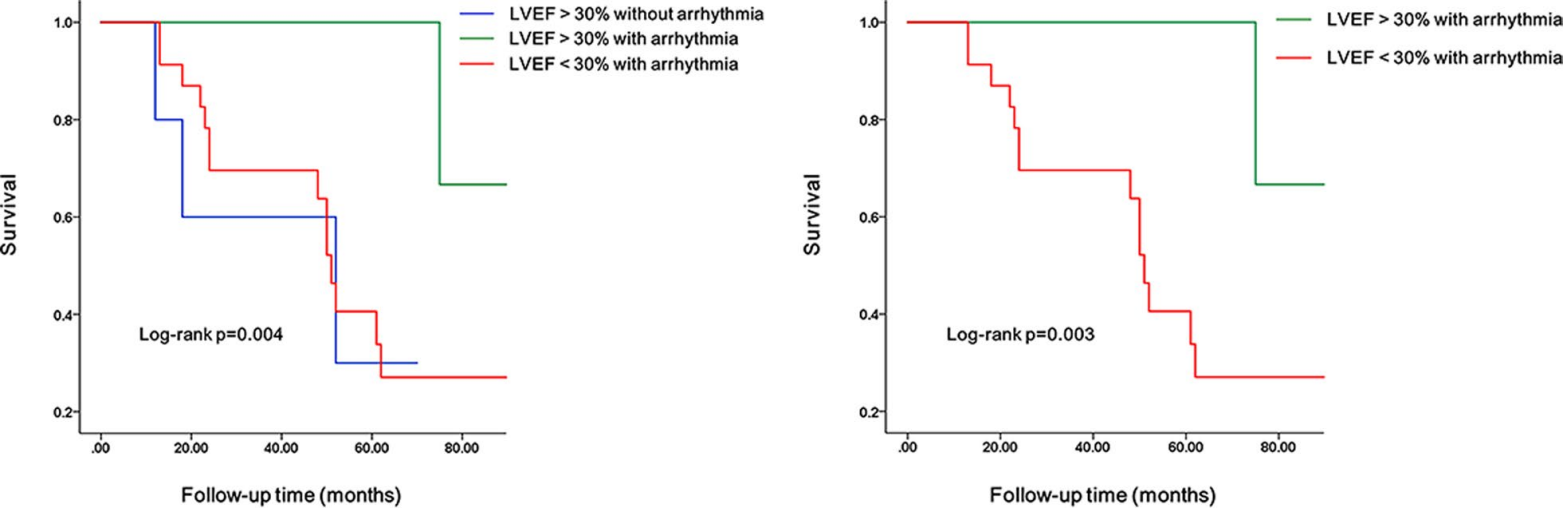
K–M survival curve incorporating LVEF into grouping conditions. Kaplan–Meier analysis among three cohorts: LVEF > 30% without arrhythmia vs. LVEF > 30% with arrhythmia vs. LVEF < 30% with arrhythmia

By analyzing the clinical and CMR characteristics of 49 LVNC patients, Kaplan–Meier analysis results showed that there were significant differences in prognosis among the three cohorts: LGE− without arrhythmia, LGE− with arrhythmia, and LGE + with arrhythmia (*p* = 0.013). LVNC patients with arrhythmia and LGE + had a worse prognosis and were more likely to have adverse cardiovascular events than LVNC patients with arrhythmia and LGE- (Fig. 4). LGE is one of the risk factors associated with the prognosis (*p* = 0.003, HR = 4.035, 95% CI 1.475–11.035). In addition, the decrease degree of LVEF may also be one of the risk factors for prognosis (*p* = 0.003, HR = 8.131, 95% CI 1.805–36.636). The difference between the other three groups, namely, the LVEF > 30% without arrhythmia group, LVEF > 30% with arrhythmia group, and LVEF < 30% with arrhythmia group, was statistically significant (*p* = 0.004). Among patients with arrhythmia, patients with more decreased LVEF had a worse prognosis (Fig. 5). The Kaplan–Meier survival analysis for arrhythmia, BMI, smoking history, alcohol consumption history, family history of cardiovascular disease, hypertension, HF, sphericity index < 1, NC/C value, and LV EDV had no significant difference.

## Discussion

In the cohort study of patients with LVNC diagnosed by CMR, we summarized the types of arrhythmias in LVNC patients, compared the CMR characteristics in patients with or without arrhythmia, and explored the association of prognosis with CMR parameters in LVNC patients with arrhythmia. We found various types of arrhythmias in LVNC patients and some types of arrhythmias with no specificity. The CMR findings showed that focal myocardial fibrosis and LGE had a higher probability of occurrence in LVNC patients with malignant arrhythmia. However, there was no significant difference among LVNC patients with different types of arrhythmias in other CMR parameters, such as LVEF. LVNC patients with arrhythmia who had LGE + or reduced LVEF had a higher incidence of adverse cardiovascular events and a worse prognosis.

Arrhythmia is one of the main clinical manifestations in LVNC patients. According to previous studies, more than 50% of LVNC patients presented with arrhythmia, which may be due to the pathological changes of myocardial tissue in LVNC patients. With the increase of myocardial cell noncompaction and trabeculation with deep intramyocardial invagination, the Purkinje system would be brought deeper into the myocardium, thus leading to both delayed depolarization and inhomogeneous repolarization [18, 19]. There are many types of arrhythmias in LVNC patients, including premature ventricular contraction, sinus tachycardia, atrioventricular block, bundle branch block, and atrial fibrillation, and ventricular arrhythmia is the most common. However, the types of arrhythmias suffered by LVNC patients had no specificity, and one patient could suffer from multiple types of arrhythmias. This finding is consistent with our results [20–22]. Some studies suggested that the prognosis of LVNC patients with arrhythmia is worse [18]. Surprisingly, our results showed that the prognosis of malignant arrhythmias did not deteriorate significantly. This result may be associated with medication. In this study, all patients received different degrees of medication, such as β-blockers and amiodarone. Therefore, drug treatment measures may effectively curb the effects of arrhythmia, reduce the impact of malignant arrhythmia on the prognosis of patients, and improve the prognosis.

The CMR findings indicated that cardiac function, such as LVEF, was generally decreased in LVNC patients compared with the healthy population, which had also been confirmed in previous reports of the patient with LVNC. Meanwhile, in previous reports, LVEF was significantly decreased with increasing noncompaction severity [23, 24]. In addition, the possibility of adverse LV remodeling was higher in LVNC patients, as evidenced by higher LV cardiac sphericity indices. This may be due to the thinning of the compaction myocardium and leads to reduced systolic function. Meanwhile, the blood supply of the heart cannot meet the blood demand of numerous myocardial trabeculae, thus leading to chronic myocardial ischemia and decreased myocardial systolic function. Given the presence of prominent myocardial trabeculae and noncompaction, irregularly distributed muscle bundles may contribute to left ventricular isomerization. However, our study found that in the cohort of LVNC patients with different degrees of arrhythmia, differences in other CMR findings were not statistically significant, except for the incidence of LGEs. The severity of arrhythmia did not further deteriorate the patient's LV function. This may be due to the fact that the changes in cardiac structure and function were mainly caused by the noncompaction of the myocardium and were less affected by arrhythmia. The results suggested that we should also pay attention to the changes in cardiac function in LVNC patients without arrhythmia or with benign arrhythmia in clinical practice.

More importantly, our results demonstrated that the incidence of LGE was different among the cohort of LVNC patients with different degrees of arrhythmias and that LVNC patients with malignant arrhythmias had a higher probability of myocardial fibrosis. Furthermore, MACEs occurred in 46.94% of patients during the follow-up. The occurrence of adverse events may be due to the abnormal myocardial structure that results in hemodynamic changes that subsequently leads to serious damage of cardiac functions [5]. At present, CMR has evolved as an effective tool for prognostic risk assessment in patients with many different forms of cardiomyopathy [25, 26], and arrhythmia is one of the most common symptoms in LVNC patients; however, there are still insufficient studies on clinical and MRI indicators for evaluating the prognosis of LVNC patients with arrhythmia, thus, we investigated the prognosis. We found that the presence of LGE was different in the cohort of patients with different degrees of arrhythmia, and LGE was one of the prognostic factors of LVNC patients with arrhythmia. The survival rate of LVNC patients with arrhythmia and presence of LGE was lower than that of patients without LGE. Jenni et al. described that coronary microcirculatory dysfunction may account for myocardial fibrosis in LVNC patients [27]. In addition, electrical inhomogeneity and the micro reentry of malignant arrhythmia were related to the relatively decreased perfusion and ischemia-related fibrosis in the subendocardial noncompacted regions [19, 28]. The disturbance of myocardial cells in LVNC patients leads to recurrent arrhythmia [29]. In LVNC patients with arrhythmias, myocardial fibrosis aggravates the damage to cardiac function, thus resulting in a more serious decline in cardiac function and leading to a poor prognosis. Fibrotic scarring was more likely to occur near the target sites of malignant ventricular arrhythmia [30]. Therefore, for LVNC patients with arrhythmia, particularly malignant arrhythmia, more attention should be paid to the occurrence of myocardial fibrosis [31]. The results emphasized the importance of the routine evaluation of myocardial fibrosis in LVNC patients with arrhythmia by using CMR-LGE, which can help evaluate myocardial fibrosis qualitatively and quantitatively and provide more information for treatment. In addition, consistent with most studies [31], the LVEF was generally reduced in LVNC patients. Some studies have shown that patients with reduced LVEF have a higher incidence of adverse events [32]. In LVNC patients with arrhythmia, we also found that a larger decrease in LVEF (LVEF < 30%) correlated with a higher incidence of adverse events. In the group with arrhythmia and with LVEF > 30%, the first adverse event occurred 72 months after the patient was diagnosed with LVNC by CMR, with no adverse events occurring before 70 months. LVNC patients with arrhythmia with slightly better cardiac function had better long-term survival outcomes than those with more reduced LVEF. Therefore, for LVNC patients with arrhythmia, the changes in LVEF should be monitored regularly, and corresponding treatment that enhances cardiac function should be adopted to improve the prognosis. The results of this study indicated that the combination of imaging indicators with arrhythmia may be more useful for risk stratification in LVNC patients.

### Limitations

Our study has several limitations. First, the number of patients in the follow-up cohort was small owing to the relatively rare patient entity. In subsequent studies, more follow-up patients need to be recruited to explore the prognostic value of different indicators in LVNC patients with arrhythmia and prospective studies are also essential. Second, LVNC patients with arrhythmia were treated with different medications at baseline, and this approach may have resulted in less significant differences in MRI performance. Third, the types of arrhythmias in LVNC patients are complex, therefore, the impact of a single arrhythmia type on the prognosis of LVNC patients and the pathophysiological mechanism of arrhythmia remains to be further studied.

## Conclusions

Arrhythmia is one of the most common clinical manifestations in LVNC patients, has many types, and has no specificity. LGE and reduced LVEF are more common in LVNC patients with malignant arrhythmia, and arrhythmia LVNC patients with LGE + or decreased LVEF have poor prognosis. The combination of imaging indicators may be more useful for risk stratification in LVNC patients.

## Data Availability

The datasets analyzed in the current study are not publicly available due to lack of consent from study participants to do so but they are available from the corresponding author on reasonable request for researchers who meet the criteria for access to confidential data.

## Abbreviations

LVNC: Left ventricular noncompaction
CMR: Cardiovascular magnetic resonance
ICD: Implantable cardioverter defibrillator
LGE: Late gadolinium enhancement
LVEF: Left ventricular ejection fraction
HF: Heart failure
VT: Ventricular tachycardia
PVC: Premature ventricular contraction
LV: Left ventricle
ECG: Electrocardiography
Vf: Ventricular fibrillation
VFL: Ventricular flutter
III°AVB: III° Atrioventricular block
WPW: Wolff–Parkinson–White syndrome
VE: Ventricular escape
SSFP: Steady-state free-precession
EDV: End-diastolic volume
ESV: End-systolic volume
SV: Stroke volume
MACEs: Major adverse cardiovascular events
CLBBB: Complete left bundle branch block
CRBBB: Complete right bundle branch block
APB: Atrial premature beats
Af: Atrial fibrillation
SVT: Supraventricular tachycardia
SVES: Superventricular premature beat
AFL: Atrial flutter
